# Disturbed Copper Bioavailability in Alzheimer's Disease

**DOI:** 10.4061/2011/345614

**Published:** 2011-11-15

**Authors:** Daniela Kaden, Ashley I. Bush, Ruth Danzeisen, Thomas A. Bayer, Gerd Multhaup

**Affiliations:** ^1^Institute of Chemistry and Biochemistry, Freie Universität Berlin, Thielallee 63, 14195 Berlin, Germany; ^2^The Mental Health Research Institute, The University of Melbourne, 155 Oak Street, Parkville, VIC 3052, Australia; ^3^International Copper Association, 260 Madison Avenue, Floor 16, New York, 10016 NY, USA; ^4^Division of Molecular Psychiatry, Georg-August-University Göttingen, University Medicine Göttingen, 37075 Göttingen, Germany

## Abstract

Recent data from in vitro, animal, and human studies have shed new light on the positive roles of copper in many aspects of AD. Copper promotes the non-amyloidogenic processing of APP and thereby lowers the A**β** production in cell culture systems, and it increases lifetime and decreases soluble amyloid production in APP transgenic mice. In a clinical trial with Alzheimer patients, the decline of A**β** levels in CSF, which is a diagnostic marker, is diminished in the verum group (8 mg copper/day), indicating a beneficial effect of the copper treatment. These observations are in line with the benefit of treatment with compounds aimed at normalizing metal levels in the brain, such as PBT2. The data reviewed here demonstrate that there is an apparent disturbance in metal homeostasis in AD. More research is urgently needed to understand how this disturbance can be addressed therapeutically.

## 1. Copper: Essential and Potentially Toxic

Copper is needed by every oxygen-requiring cell and can be toxic in excess. It is an essential metal with extremely complex roles in numerous different biological functions from acute phase reactant to mitochondrial energy generation. Cu levels are very tightly regulated on the level of duodenal absorption as well as uptake into cells or excretion from cells. Intracellularly, it is transported by specialized chaperone-like proteins to protect the ion from reactions with reactive oxygen species [1]. 

Plasma Cu levels do not correspond to an individual's Cu exposure or Cu status. Cu serum levels rise during acute phase, they correlate positively with estrogen levels, and can also rise during many chronic disease states. This is caused by an increase in a Cu-containing plasma protein called ceruloplasmin (Cp). Most of the plasma Cu is bound to Cp (up to 95%), with the remaining portion of the Cu being bound to albumin or histidine residues in other proteins [2]. 

A possible role of Cu in AD has remained a contentious topic during the past 15 years, as has been the question whether extracellular amyloid deposited in plaques is the causative agent in AD. The scientific community was divided as to whether Cu has a role at all, and—if yes—whether it is friend or foe. 

Intensive basic research has led to a paradigm shift in both unveiling a beneficial role for Cu in AD on the one hand and deleterious functions of lower intracellular amyloid oligomers consisting of the 42 amino acid residue amyloid-*β* peptide (A*β*42).

## 2. Copper and Alzheimer's Disease: Molecular and In Vitro Findings

On a molecular level, interactions between Cu and proteins involved in AD are observed, that is, the amyloid precursor protein (APP), the beta-site APP-cleaving enzyme 1 (BACE1), and the A*β* peptide. APP has a role in Cu-efflux [3] and binds Cu(II) via its extracellular domain [4, 5], where bound Cu(II) is reduced to Cu(I) [6, 7]. Additionally, APP trafficking is directly influenced by Cu. Cu treatment of neuronal cells revealed an increase of APP at the cell surface by both promoting its exocytosis from the Golgi and by reducing its rate of endocytosis [8, 9]. BACE1 is an aspartic protease, cleaving APP in the first step of A*β* generation. BACE1 binds a single Cu(I) atom with high affinity through cysteine residues of its C-terminal domain and interacts with the cytoplasmic Cu-chaperone CCS (the Cu chaperone for Cu, Zn-superoxide dismutase (SOD1)) through domain I [10]. A*β*, which is part of the ectodomain and the transmembrane segment of APP, was also shown to bind Cu, and Cu was found enriched in plaque deposits which mainly consist of A*β* [11–14]. Thus, APP, BACE1, and A*β* are metalloproteins which can bind Cu and were experimentally shown to be involved in brain Cu homeostasis. 

In 1994, the aggregation of synthetic A*β* peptides into assemblies of oligomers and fibrils upon binding of Cu(II) was reported [15]. It was the time before the toxicity of lower A*β* oligomers was discovered and insoluble plaque amyloid was found inert [16]. Over the years, the hypothesis of oligomers as causative agents was confirmed [17]. Recent research has provided evidence that oligomers were shown to induce hyperphosphorylation of tau at AD-relevant epitopes in hippocampal neurons, and thereby provided a strong link between the pathological hallmarks of A*β* and tau deposits [18]. Oligomers isolated from the AD brain were found to potently induce AD-type tau phosphorylation. Tau was found to form Cu-complexes with one Cu(II) ion bound per monomer with a dissociation constant in the micromolar range Cu(II), having an inhibiting effect on the in vitro aggregation of tau [19, 20]. However, since tau occurs intracellularly and Cu(II) is mainly found in the extracellular space, the likelihood that this reflects a physiological function is low.

Findings such that human A*β* directly produces hydrogen peroxide (H_2_O_2_) by a mechanism that involves the reduction of metal ions, Fe(III) or Cu(II), setting up conditions for Fenton-type chemistry [21] or that Cu(II) and Zn(II) inhibit A*β* fibrillization [22, 23] were all generated by in vitro assays and were never systematically addressed by in vivo model systems.

## 3. Copper and Alzheimer's Disease: Findings in Animal Models

Maynard et al. have shown that overexpression of the carboxy-terminal fragment of APP which contains the A*β* sequence elicits significantly reduced Cu levels in transgenic mouse brains [24]. Importantly, animal model systems of AD and studies with living cells revealed that APP is actively involved in balancing Cu concentrations. In APP and APLP2 knockout mice, Cu levels were found increased in cerebral cortex and liver [25], whereas overexpression of APP was reported to result in significantly reduced Cu levels in the brain of three transgenic mouse lines [24, 26]. 

APP23 mice (expressing human APP751 with the Swedish mutation) have amyloidogenic deposits after 6 months and more than half of the population dies before the age of 18 months. Treatment with clioquinol, a hydrophobic low-affinity Cu, and zinc chelator had a significant benefit in this AD mouse model by normalizing, which is elevating, brain Cu and zinc levels. In the treated animals brain, A*β* deposition was decreased, and the general health of the animals was improved [27]. 

A later study in two different strains of amyloid-bearing transgenic mice confirmed the cognitive benefits of clioquinol treatment [28]. Treatment with clioquinol‘s second-generation analogue, PBT2, rapidly restored cognition disturbances in AD transgenic mice and was associated with decreased interstitial A*β* [29]. Further, the neurotrophic effects of PBT2 have recently been demonstrated to depend upon metal uptake [30].

Consistent with the above findings, a dietary treatment of APP23 transgenic mice with Cu sulfate for a 3-month interval extended the lifetime of the mice considerably and restored SOD-1 activity back to normal [26]. In agreement with the benefit of Cu treatment, Cu supplementation rescued premature death observed with clioquinol treatment of APP transgenic mice [31]. This is in contrast, however, to the above findings of benefits of clioquinol and PBT2 treatment. 

Taken together, these observations indicate that restoring brain Cu homeostasis can have a beneficial effect on disease progression in mouse models for AD. Cu levels can be normalized by dietary treatment with bioavailable Cu salts, or by treatment with clioquinol, which normalizes Cu and zinc brain levels through the formation of metal-ion complexes which are transported across cellular membranes. 

In contrast, another animal model, hypercholesterolemic rabbits, showed amyloid-inducing effects mediated by low Cu concentrations in drinking water (0.12 mg Cu/L) [32–34]. This effect was observed when Cu sulfate was added to distilled water, and not when tap water was used in combination with the high cholesterol feed [32]. Potable tap water typically contains at least this amount of Cu (0.12 mg Cu/L), present as Cu carbonate or Cu carbonate hydroxide. Also, when interpreting the findings in the hypercholesterolemic animals, it needs to be kept in mind that high cholesterol levels itself show A*β*-elevating effects [35].

## 4. Copper and Alzheimer's Disease: Human Trials

Based on the animal study [26], oral intake of Cu(II) in AD patients was investigated in a clinical trial. The efficacy of oral Cu supplementation in the treatment of AD patients was evaluated for 12 months in a prospective, randomized, double-blind, placebo-controlled phase 2 clinical trial in patients with mild AD. Sixty-eight subjects were randomized. Patients with mild AD received either Cu-(II)-orotate-dihydrate (verum group; 8 mg Cu daily) or placebo (placebo group). CSF was collected at beginning and at the end of the study after 12 months. The treatment was well tolerated. The primary outcome measures in CSF were A*β*42, Tau, and Phospho-Tau. Cu intake had no effect on the progression of Tau and Phospho-Tau levels in CSF [36]. 

While A*β*42 levels declined by 30% in the placebo group (*P* = 0.001), they decreased only by 10% (*P* = 0.04) in the verum group [36]. Since decreased CSF A*β*42 is a diagnostic marker for AD, this observation indicated that Cu treatment had a positive effect on a relevant AD biomarker. There were, however, no significant differences in primary outcome measures (AD Assessment Scale, Cognitive subscale (ADAScog), Mini Mental Status Examination) between the verum and the placebo group [37]. Finally, CSF A*β*42 levels declined significantly in both groups within 12 months supporting the notion that CSF A*β*42 may be valid not only for diagnostic but also for prognostic purposes in AD [36]. 

Plasma Cu levels declined only in the placebo group during the 12-month period. However, the outcome of the randomization was such that the placebo group had higher Cu levels at the beginning of the study. Cu levels in the verum group were unchanged, which seems to be paradoxical. One may speculate that Cu treatment normalized Cu levels in plasma in the verum group by enhanced uptake and transport and improved tissue homeostasis.

Previously, we have reported that significantly lower levels of Cu in plasma were found in those AD patients, who fulfilled the criteria of CSF diagnosis for AD [38]. In addition, we observed reduced Cu levels in plasma in patients with higher ADAScog scores (making more mistakes in this neuropsychological test) [39]. However, Cu treatment had no beneficial effect on cognitive abilities in AD patients of the present clinical phase II pilot study [37]. There was no correlation between plasma or CSF Cu and cholesterol levels. 

The Cu-clinical trial demonstrates that long-term oral intake of 8 mg Cu (Cu-(II)-orotate-dihydrate) can be excluded as a risk factor for AD, and—based on the CSF biomarker analysis—that Cu may potentially play a beneficial role in this disease.

This is consistent with findings from a pilot phase II clinical trial, where the placebo group deteriorated faster than the clioquinol-treated group suggesting a beneficial effect of clioquinol treatment [40]. In line with these observations, treatment of AD patients with PBT2 in a phase IIa, double-blind, randomized, placebo-controlled trial, PBT2 induced a significant improvement in cognitive performance after 12 weeks without changing plasma Cu levels [41]. 

Although we found beneficial effects of Cu, there are contradicting results showing that elevated free serum Cu levels (non-ceruloplasmin bound Cu) might be a risk factor for AD [42–47]. A high proportion of the ceruloplasmin of these patients was enzymatically inactive [47], indicating that it contained less-than-normal Cu. Ceruloplasmin is the main Cu carrier in plasma and has many roles, such as ferroxidase or acute phase reactant. However, ceruloplasmin is not a prerequisite for Cu delivery to the periphery [48]. The studies by Squitti, Arnal, and Brewer evaluating levels of non-ceruloplasmin Cu in AD patients do not indicate the chemical nature of this Cu. It is, therefore, unclear whether or not this Cu is readily bioavailable or not. In any case, inactive ceruloplasmin and potentially less bioavailable “free” Cu will directly influence (i.e., lower) intracellular Cu levels and impair the functions of proteins using Cu as a cofactor.

“Free” Cu can have negative effects in the brain as seen in disease states like Wilsons disease. However, levels of Cu in CSF were not upregulated in AD as revealed in a metaanalysis [49]. Instead, the study of Kessler et al. revealed reduced plasma Cu and ceruloplasmin levels in patients with a CSF diagnosis of advanced AD which supports previous observations that a mild Cu deficiency might contribute to AD progression [38]. 

Whether the observed increase in the non-ceruloplasmin Cu portion is a cause or consequence of the AD pathogenesis also remains to be clarified. The conclusion that Cu intake or exposure can be a risk factor for AD cannot be drawn and a direct comparison between the work by Squitti et al and Brewer et al with an intervention study (8 mg Cu orotate per day, or treatment with PBT2) is difficult.

## 5. Strategies to Rescue Copper Deficiency: Chelators, Ionophores, and Cu Nanocarriers

APP and APLP2 extracellular domains, but not the extracellular domain of APLP1, decreased intracellular Cu levels in yeast cells and thus possess Cu-efflux activities in this test system [3]. The addition of clioquinol-Cu complexes to the yeast culture medium drastically increased the intracellular Cu concentration, but there was no significant effect on zinc levels. This finding confirmed that facilitated metal-ion transport can act therapeutically by changing the distribution of Cu or facilitating Cu uptake rather than by decreasing Cu levels. 

The expression of a mutant APP deficient for Cu binding increased intracellular Cu levels several fold [3]. These data not only uncovered a novel biological function for APP and APLP2 in cellular Cu homeostasis but also provided a new conceptual framework for the formerly diverging theories of Cu supplementation and chelation in the treatment of AD [27]. These results also strictly contradict a proposed metal chelation as a potential therapy for AD that was based on the A*β* interaction with transition metals [50].

Findings described above [3] and in the section of findings from animal models [31] encouraged us to address Cu-deficiency in AD by an aimed facilitation of Cu import into the brain of Cu deficient AD patients. Thus, several parameters have to be taken into consideration, and several requirements must be regarded. The transport and cellular metabolism of Cu depends on a series of membrane proteins and smaller soluble proteins that comprise a functionally integrated system for maintaining cellular Cu homeostasis. 

Bypassing the cellular Cu uptake system would be a mean to achieve higher local concentrations compared to normal cellular uptake. We were able to show that synthetic substances called nanocarriers fulfill these requirements. They specifically transport Cu to intracellular regions and enrich local Cu concentrations (e.g. in endosomes versus cytosol) [51]. Cu released from the carrier was bioavailable and compensated decreased Cu levels in living cells. We and others showed earlier that high intracellular Cu levels stimulate the non-amyloidogenic pathway of APP processing, thereby, diminishing levels of toxic A*β* peptides [52, 53]. Vice versa, under conditions of low intracellular Cu, APP proteolysis was shifted from non-amyloidogenic to amyloidogenic processing [52, 54].

Taken together, Cu has beneficial roles in the course of AD based on the following observations: (i) it promotes the non-amyloidogenic processing of APP and thereby lowers the A*β* production in cell culture systems, (ii) it increases lifetime and decreases soluble amyloid production in APP transgenic mice, and (iii) in a clinical trial with AD patients, the decline of A*β* levels in CSF, which is a diagnostic marker, is diminished. More research is urgently required to understand why there is an apparent disturbance in metal homeostasis in AD and how this disturbance can be addressed therapeutically.

## Abbreviations

AD: Alzheimer's disease
APP: Amyloid precursor protein
BACE1: Beta-site APP cleaving enzyme 1
Cp: Ceruloplasmin
Cu: Copper
PBT2: Second-generation 8-hydroxy quinoline analog
SOD1: Cu, Zn superoxide dismutase
ADAScog: AD Assessment Scale, Cognitive subscale.
